# A novel splice site variant in *CYP11A1* in *trans* with the p.E314K variant in a male patient with congenital adrenal insufficiency

**DOI:** 10.1002/mgg3.322

**Published:** 2017-07-20

**Authors:** Montserrat Lara‐Velazquez, Alexander Perdomo‐Pantoja, Patrick R. Blackburn, Jennifer M. Gass, Thomas R. Caulfield, Paldeep S. Atwal

**Affiliations:** ^1^ Department of Neurosurgery Mayo Clinic Jacksonville Florida; ^2^ Department of Neurosurgery Johns Hopkins University Baltimore Maryland; ^3^ Center for Individualized Medicine Mayo Clinic Jacksonville Florida; ^4^ Department of Health Sciences Research Mayo Clinic Jacksonville Florida; ^5^ Department of Neuroscience Mayo Clinic Jacksonville Florida; ^6^ Department of Clinical Genomics Mayo Clinic Jacksonville Florida

**Keywords:** c.425+1G>A variant, *CYP11A1*, primary adrenal insufficiency

## Abstract

**Background:**

The *CYP11A1* gene encodes the cytochrome P450 side‐chain cleavage enzyme, which is essential for steroid formation. Recessive variants in this gene can lead to impairment of sexual differentiation caused by a complete or partial loss of steroid hormone production. The phenotypic spectrum in affected 46XY males may vary from surgically repairable defects including cryptorchidism and hypospadias to complete feminization of external gonads, accompanied by symptoms of adrenal dysfunction.

**Methods:**

Whole‐exome sequencing (WES) of a 12‐year‐old male proband and his parents was performed after a protracted diagnostic odyssey failed to uncover the cause of his primary adrenal insufficiency. Of note, the proband had early symptomatology and corrective surgery for hypospadias, raising suspicion for a disorder of steroidogenesis.

**Results:**

WES identified compound heterozygous variants in *CYP11A1* including a novel canonical splice site variant (c.425+1G>A) and a previously reported p.E314K variant, which were consistent with a diagnosis of congenital adrenal insufficiency with partial 46XY sex reversal.

**Conclusion:**

Congenital adrenal insufficiency with 46XY sex reversal is a rare disorder that is characterized by dysregulation of steroid hormone synthesis, leading to adrenal and gonadal dysfunction. In this report, we describe a patient with adrenal insufficiency, hypospadias, and skin hyperpigmentation who was found to have a novel c.425+1G>A variant in *trans* with the p.E314K variant in *CYP11A1*. We performed structural analyses to examine the effect of the p.E314K variant on protein function and show that it falls in the core of the protein may disrupt cholesterol binding in the active site.

## Introduction

The *CYP11A1* gene located in the long arm of chromosome 15 (15q23–q24) encodes the cholesterol side‐chain cleavage enzyme which converts cholesterol to pregnenolone, the first step for corticoid, mineralocorticoid, and sexual hormone biosynthesis (Miller and Auchus 2011). CYP11A1 deficiency results in complete or partial adrenal insufficiency with a wide range of clinical manifestations. Severe or classical CYP11A1 deficiency manifests with feminization of external genitalia in 46XY males and severe early onset adrenal failure in the first few hours or days of postnatal life. Mild or nonclassical CYP11A1 deficiency is associated with adrenal insufficiency that can develop later in childhood and is accompanied by a spectrum of sexual development disorders including precocious or delayed puberty (Katsumata et al. 2002; Hiort et al. 2005; Slominski et al. 2015).

### Conclusion

In this report, we present a case of a 12‐year‐old male with congenital adrenal insufficiency. Our patient began having symptoms at 3 years old, which included lethargy, hypoglycemia, hyperpigmentation, hypospadias, and biochemical findings that confirmed adrenal insufficiency. Clinical WES revealed compound heterozygous variants in *CYP11A* including a novel c.425+1G>A splice site mutation and a previously reported missense variant (p.E314K). To our knowledge, this splice site variant has not been reported previously but is likely pathogenic and the cause of our patient's primary adrenal insufficiency.

## Case Description

The patient is a 12‐year‐old male, who is the second child of nonconsanguineous parents. He was delivered via vaginal route after induction of labor on the 34th week of pregnancy due to HELLP (Hemolysis, Elevated Liver enzymes, Low Platelet count) syndrome and toxemia and was in the neonatal intensive care unit for 3 weeks for recovery and treatment of neonatal jaundice. His birth weight and length were 2.24 kg and 47 cm, respectively. Upon examination of the external genitalia, hypospadias were detected and later surgically repaired; cryptorchidism was not a feature.

At 3 years of age, he began having severe diurnal episodes of lethargy and hypoglycemia. He was also noted to have skin hyperpigmentation. Testing of hormone levels revealed high adrenocorticotropic hormone (ACTH) and low cortisol levels, suggestive of a primary adrenal insufficiency. An ultrasound and CT scan of the adrenal glands at 3 years of age did not show abnormal findings. Genetic testing for the *NR0B1* (DAX1), which was involved in X‐linked congenital adrenal hypoplasia (MIM# 300200), was negative. At 8 years of age, his bloodwork showed an ACTH of 6 pg/mL (normal range 10–60 pg/mL), 17‐hydroxyprogesterone of ˂10 ng/dL (Prepubertal males: <110 ng/dL), and testosterone levels below 2.5 ng/dL (normal rage for males 6 months to 9 years: <7–20 ng/dL). By 9 years of age, his ACTH was abnormally high at 261 pg/mL. Then at 10 years of age, his ACTH level increased to 321.9 pg/mL and repeat testing a year later revealed levels of 405 pg/mL. During his last medical visit, no signs of altered puberty were noticed, and initiation of corticosteroid treatment lead to the normalization of ACTH levels (32.4 pg/mL post treatment). This also improved his hyperpigmentation. No investigations were performed for precocious puberty as his growth and development were age appropriate. He did have a bone age, however, at age 8 9/12 which showed a bone age of 8. The patient was also diagnosed with attention‐deficit/hyperactivity disorder (ADHD) and migraine. Currently, he is under treatment with hydrocortisone (10 mg/m^2^/day in the mornings and 5 mg/m^2^/day at night), fludrocortisone (50 μg/day), amphetamine‐dextroamphetamine (15 mg/day), and vitamin D (1000 units/day).

**Figure 1:**
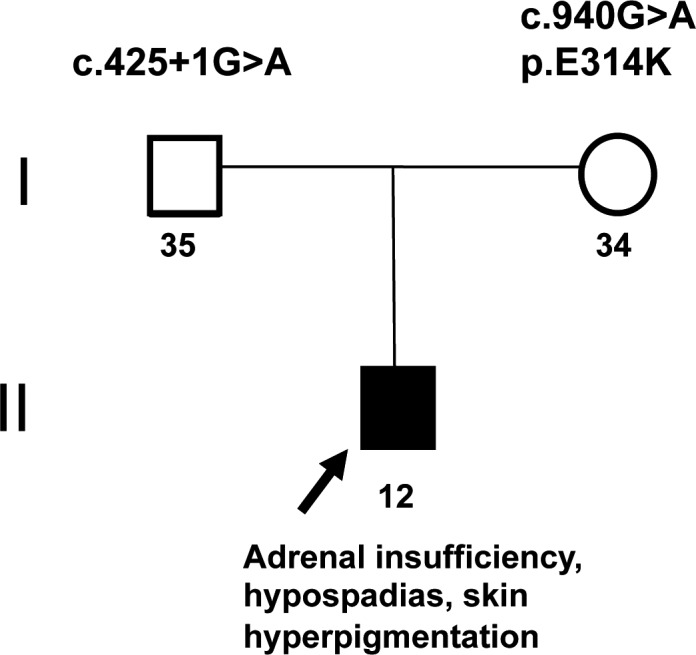
Patient family history. Black arrow is depicting the affected subject. Patient was diagnosed with hypospadias and skin hyperpigmentation. There was no family history of adrenal insufficiency.

The patient presented to the Department of Clinical Genomics (Mayo Clinic, Jacksonville, FL) with the finding of primary adrenal insufficiency of unknown genetic cause. Informed consent was obtained and blood samples were collected from the patient and his parents for WES. There was no family history of adrenal insufficiency or urogenital anomalies (Fig. 1). Previous genetic testing included mitochondrial DNA (mtDNA) sequencing which revealed no relevant pathogenic variants.

## Materials and Methods

### Ethical compliance

Our study was approved by Mayo Clinic IRB and ethics committees.

### Testing

In order to identify the genetic etiology, WES was performed. The patient and his family were counseled about the purpose, benefits, and limitations of WES. Additionally, possible test results were reviewed with patient and parents. The protections and limitations of GINA (Genetic Information Nondiscrimination Act) were discussed. The patient's mother was provided a copy of the informed consent. Samples from the proband, mother, and father were collected. Due to the history of HELLP syndrome in the patient's mother, acylcarnitine testing was also performed in the proband, which was normal.

### Whole‐exome sequencing

Clinical WES was performed by GeneDX (XomeDxPlus). Briefly, genomic DNA was extracted from the proband and parental samples. As described in the clinical testing methodology by GeneDX, the Agilent Clinical Research Exome capture kit was used for exome enrichment and sequencing was done on an Illumina HiSeq 2000 that generates 100 bp paired‐end reads. Bidirectional sequence was assembled, aligned to reference gene sequences based on human genome build GRCh37/UCSC hg19, and analyzed for sequence variants using a proprietary analysis tool (Xome Analyzer, GeneDx). Sanger sequencing was used to confirm all potentially pathogenic variants identified in this individual. Sequence alterations were reported according to the Human Genome Variation Society (HGVS) nomenclature guidelines.

### Molecular modeling

The sequence of human cholesterol side‐chain cleavage enzyme, mitochondrial 11A1 (Cyp11A1), a protein encoded by the gene (*CYP11A1*), was taken from the NCBI Reference Sequence: NM_000781.2: NP_000772.2; and was used for modeling. Monte Carlo simulations were performed on the mutant to allow local regional changes after the p.E314K variant was introduced. Additional details can be found in the Appendix S1.

## Results

WES revealed compound heterozygous variants in the proband, including a novel paternally inherited *CYP11A1* splice site variant (Chr15(GRCh37): g.74640240C>T, NM_000781.2: c.425+1G>A) and a previously reported *CYP11A1* missense variant (Chr15(GRCh37): g.74635368C>T, NM_000781.2: c.940G>A) that was maternally inherited (Table 1). The c.425+1G>A variant is considered pathogenic and the c.940G>A (p.E314K) variant likely pathogenic based on HGVS guidelines. Variants were deposited in ClinVar under accession numbers RCV000403766.1 and RCV000413593.1, respectively.

**Table 1:** Causative variants in disease genes associated with reported phenotype

CYP11A1 phenotype	MOI	Variant	Variant location	Zygosity	Inheritance	Classification
Adrenal insufficiency, congenital, 46XY sex reversal, partial or complete (MIM #613743)	AR	IVS2+1G>A	NP_000772.2	Heterozygous	Father	Pathogenic
		c.425+1G>A	NM_000781.2			
		g.74640240C>T	NG_007973.1			
	AR	p.Glu314Lys	NP_000772.2	Heterozygous	Mother	Likely pathogenic
		c.940G>A	NM_000781.2			
		g.74635368C>T	NG_007973.1			

## Discussion

Adrenal insufficiency (AI) is characterized by defects in cortisol, aldosterone, and sexual hormone production (Miller 2017). Common manifestations of the disease include hyperpigmentation, hypoglycemia, lethargy, fatigue, muscle weakness, weight loss, dizziness and hypotension (Ucar et al. 2016). Biochemical criteria for AI diagnosis include very low serum cortisol levels (<80 nmol/L), and elevated ACTH plasma levels (>200 pmol/L) (Neary and Nieman 2010; Ucar et al. 2016).

**Figure 2:**
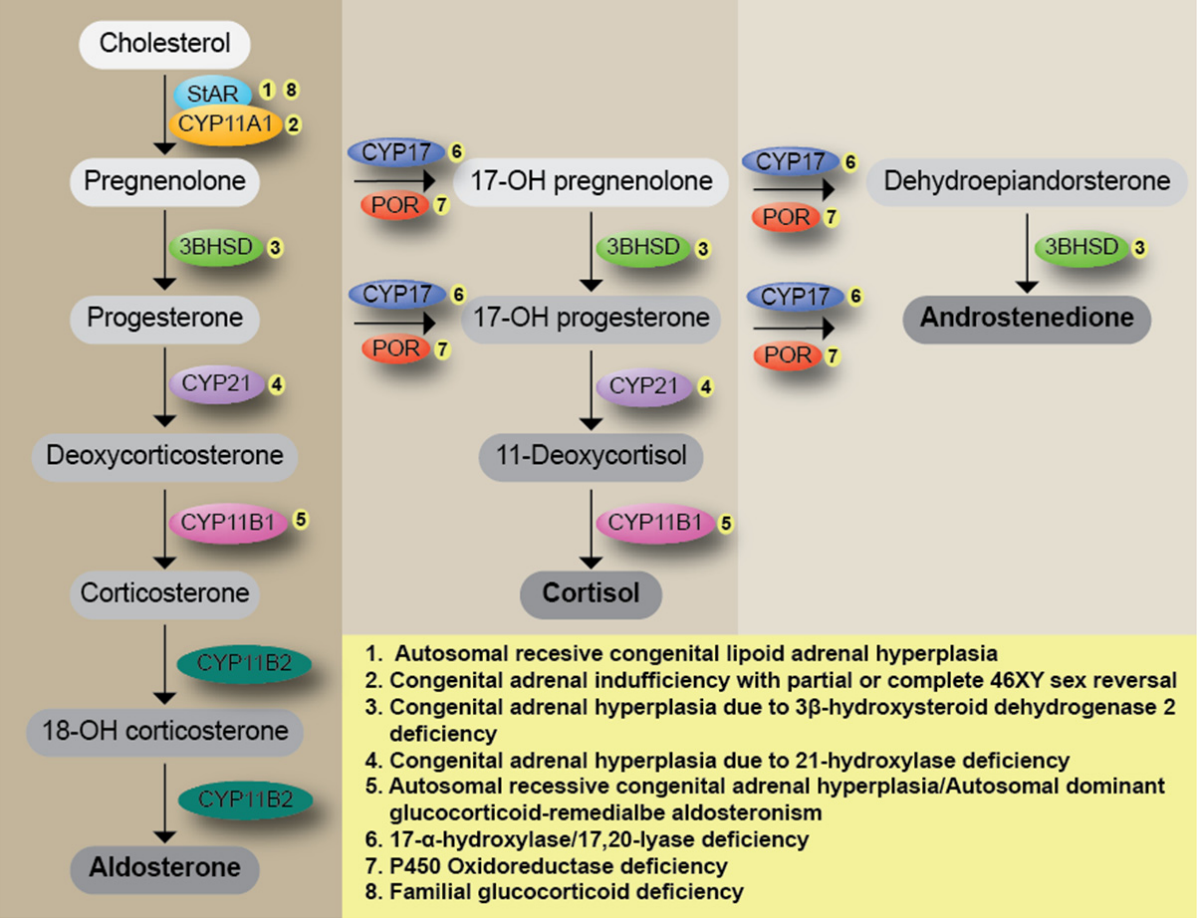
Steroid hormone synthesis diagram and related pathologies (1–3).

AI can be produced either by congenital or acquired pathologies. Congenital causes include autosomal recessive congenital lipoid adrenal hyperplasia (CAH, MIM# 201710) (caused by pathogenic variants in *STAR*); congenital adrenal insufficiency with partial or complete 46XY sex reversal (AICSR, MIM# 613743) (caused by variants in *CYP11A1*), congenital adrenal hyperplasia due to 3*β*‐hydroxysteroid dehydrogenase 2 deficiency (caused by variants in *HSD3B2*); congenital adrenal hyperplasia due to 21‐hydroxylase deficiency (MIM# 201910) (caused by variants in *CYP21A2*); 11*β*‐hydroxylase deficiency (caused by variants in *CYP11B1*) which can result in autosomal recessive congenital adrenal hyperplasia (MIM# 202010) or autosomal dominant glucocorticoid‐remediable aldosteronism (MIM# 103900); 17‐*α*‐hydroxylase/17,20‐lyase deficiency (MIM# 202110) (caused by variants in *CYP17A1*); P450 oxidoreductase deficiency (MIM# 201750) (caused by variants in *POR*) (Katsumata et al. 2002; Hiort et al. 2005; Slominski et al. 2015); familial glucocorticoid deficiency (FGD) (caused by defects in *STAR*,* MC2R*,* MRAP*,* MCM4*,* NNT*,* TXNRD2*,* GPX1* and *PRDX3*) (Miller 2017); and autoimmune disorders such as polyendocrinopathy syndrome type I, with or without reversible metaphyseal dysplasia (MIM# 240300, caused by defects in *AIRE*) (Fig. 2) (Miller and Auchus 2011; Turcu and Auchus 2015; Manna et al. 2016). Acquired causes, include infectious diseases (Waterhouse‐Frederichsen syndrome), vascular diseases (hemorrhage and coagulopathy), surgery (bilateral adrenalectomy), and exposure to certain medications (rifampicin, phenytoin, and ketoconazole) (Zaloga and Marik 2001).

Congenital AI is an autosomal recessive disorder characterized by elevated ACTH and plasma renin levels with low or absent adrenal steroids. AI has two different expression forms: classical and nonclassical (Pomahacova et al. 2016). The classical phenotypic spectrum ranges from miscarriage and prematurity during pregnancy, to complete under‐androgenization and severe early or late onset adrenal failure at postnatal stages. In the classic presentation of the disease, clitoromegaly is a common finding for feminization of external genitalia in 46XY patients (Neary and Nieman 2010). In the nonclassic form, patients can present with adrenal function impairment with minimal effects on primary sexual characteristics and male patients may present with only cryptorchidism or hypospadias (Hauffa and Hiort 2011; Tee et al. 2013).

**Figure 3:**
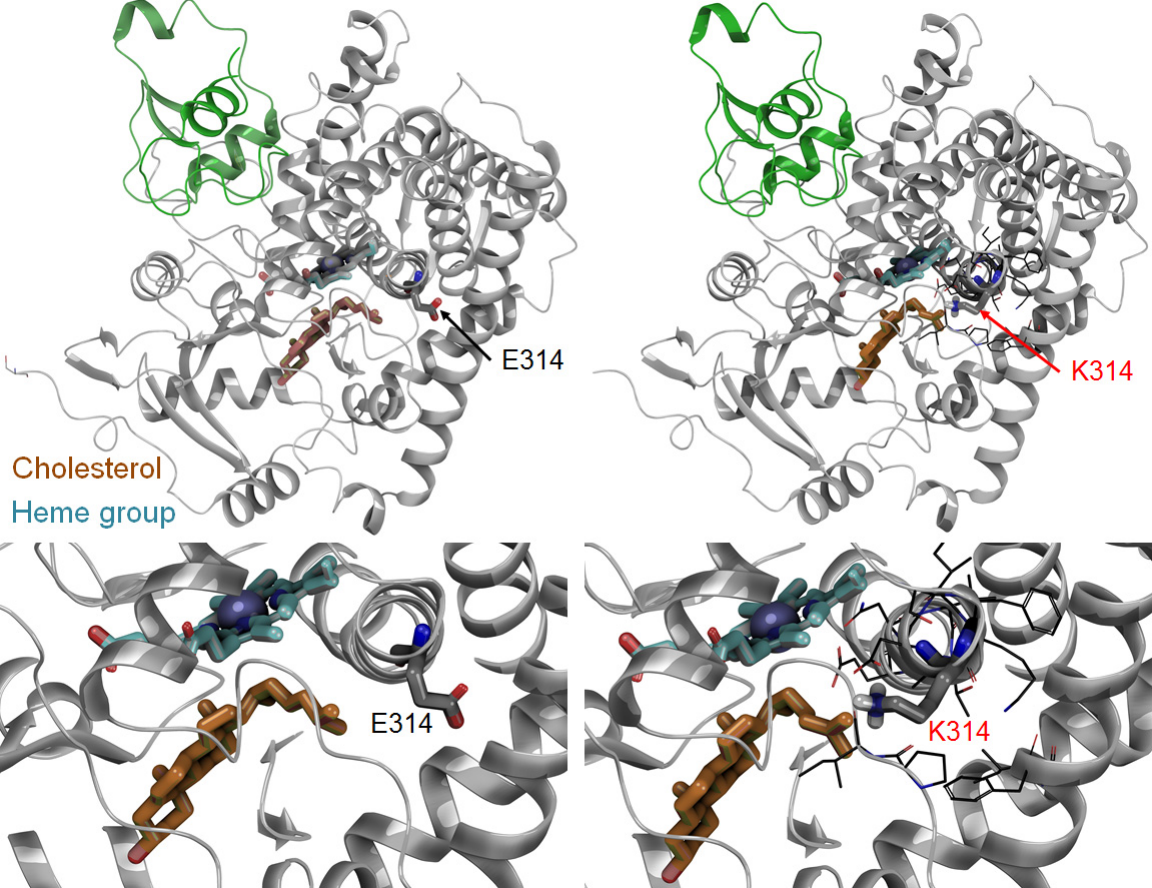
Schematic representation of P450 side‐chain cleavage (CYP11A1) and its interaction with the E314K variant.

Adrenal insufficiency can be caused by pathogenic variants in *CYP11A1*. *CYP11A1* is located at chromosome 15q23–24, and encodes the cytochrome P450 side‐chain cleavage enzyme (P450scc), which initiates steroidogenesis (Fig. 3). P450scc catalyzes the conversion of cholesterol to pregnenolone and isocaproic aldehyde in the inner mitochondrial membrane. These reactions represent the first step for steroid‐hormone biosynthesis. Biallelic pathogenic variants in *CYP11A1* are a rare cause of adrenal insufficiency in patients (Tajima et al. 2001; Hauffa and Hiort 2011; Parajes et al. 2011, 2012; Tee et al. 2013). Compared with CAH, AI patients can present mild to severe adrenal insufficiency symptoms without adrenal hyperplasia, these symptoms can manifest at any time from infancy to early childhood, depending on the degree of P450scc dysfunction (Hauffa and Hiort 2011; Tee et al. 2013).

In this report, we found a c.425+1G>A pathogenic variant in *CYP11A1* in our 12‐year‐old male patient. This splice site variant disrupts the canonical splice donor site in intron 2, and is predicted to cause abnormal gene splicing. The c.425+1G>A variant is a rare variant and has not been seen in NHLBI Exome Sequencing Project or the Genome Aggregation Database (gnomAD) (insert database references) (Table 1). To the best of our knowledge, the c.425+1G>A pathogenic variant in *CYP11A1* has not been reported previously. Regarding ACTH levels, records indicate no cosyntropin stimulation test was performed, and perhaps would have led to earlier diagnosis of adrenal insufficiency and initiation of steroids.’

In addition to the c.425+1G>A variant, we identified a missense variant (c.940G>A p.(E314K)) in exon 5. The p.(E314K) variant is a nonconservative amino acid substitution, which likely impacts secondary protein structure as these residues differ in polarity, charge, and size. However, this substitution occurs at a position that is not conserved, and in silico analysis is inconsistent in its predictions as to whether or not the variant is predicted to be damaging. The p.(E314K) variant is a strong candidate for a pathogenic variant. In two unrelated individuals with primary adrenal insufficiency, the p.(E314K) variant was previously reported; one of this cases was accompanied by another *CYP11A1* variant *in trans* with another variant whereas the other reported case had no additional molecular findings (Chan et al. 2015). According with the NHLBI ESP Exome Sequencing Project, p.(E314K) variant is present in 29/8592 cases from individuals of European American ancestry and in 710/277190 individuals in gnomAD, including four homozygotes, (Table 1) suggesting that this variant may retain partial function and could result in disease when *in trans c*with another inactivating mutation. Molecular modeling of the p.E314K variant was performed and Monte Carlo simulations suggest that the orientation of the E and K residues flips and the mutant may disrupt cholesterol binding and lead to minor changes in helicity at the end of the helix when K314 is introduced (Fig. 3).

## Conclusion

In conclusion, the compound heterozygous presence of the c.425+1G>A and p.(E314K) variants in *CYP11A1* found in this patient, explain the reported primary adrenal insufficiency diagnosis. The c.425+1G>A splice variant in *CYP11A1* is novel and may be a pathogenic variant for AI, the p.(E314K) variant has been cited in the literature as pathogenic when present as a compound heterozygous variant (Chan et al. 2015). In addition to the novel variant description, this case remarks the application of WES as a powerful diagnostic tool to provide accurate diagnosis, medical management and family counseling to improve patient care.

## Conflict of Interest

The authors declare no conflicts of interest.

## Authors’ Contributions

M.L.V., A.P.P., P.R.B., and P.S.A. designed the study and wrote the manuscript. T.R.C. performed the in silico analyses. P.R.B. and J.M.G. assisted with data collection and provided critical review of the manuscript. P.S.A. collected the clinical data. All authors approved the final version of the manuscript for publication.

## Supporting information


**Appendix S1.** Methods: Computer‐assisted modeling of CYP11A1 protein structure.
