# Supplementary material for: A novel splice site variant in CYP11A1 in trans with the p.E314K variant in a male patient with congenital adrenal insufficiency
Source: Mol Genet Genomic Med. 2017 Jul 20;5(6):781–7. doi: 10.1002/mgg3.322 (PMC5702577; doi:10.1002/mgg3.322)
Supplement: Supplementary file 1 — Appendix S1. Methods: Computer‐assisted modeling of CYP11A1 protein structure. [file MGG3-5-781-s001.doc]

**Supplementary Methods:**

**Computer assisted modeling of CYP11A1 protein structure**

CYP11A1 is a cholesterol side-chain cleavage enzyme, mitochondrial protein, which catalyzes the side-chain cleavage reaction of cholesterol to pregnenolone. The sequence of human Cholesterol side-chain cleavage enzyme, mitochondrial 11A1 (Cyp11A1), a protein encoded by the gene (CYP11A1), was taken from the NCBI Reference Sequence: NM_000781.2: NP_000772.2; and the following 521 amino acid (aa) residues from the FASTA sequence used for modeling: MLAKGLPPRSVLVKGCQTFLSAPREGLGRLRVPTGEGAGISTRSPRPFNEIPSPGDNGWLNLYHFWRETGTHKVHLHHVQNFQKYGPIYREKLGNVESVYVIDPEDVALLFKSEGPNPERFLIPPWVAYHQYYQRPIGVLLKKSAAWKKDRVALNQEVMAPEATKNFLPLLDAVSRDFVSVLHRRIKKAGSGNYSGDISDDLFRFAFESITNVIFGERQGMLEEVVNPEAQRFIDAIYQMFHTSVPMLNLPPDLFRLFRTKTWKDHVAAWDVIFSKADIYTQNFYWELRQKGSVHHDYRGILYRLLGDSKMSFEDIKANVTEMLAGGVDTTSMTLQWHLYEMARNLKVQDMLRAEVLAARHQAQGDMATMLQLVPLLKASIKETLRLHPISVTLQRYLVNDLVLRDYMIPAKTLVQVAIYALGREPTFFFDPENFDPTRWLSKDKNITYFRNLGFGWGVRQCLGRRIAELEMTIFLINMLENFRVEIQHLSDVGTTFNLILMPEKPISFTFWPFNQEATQQ. The protein has well characterized secondary structure within the X-ray structure; Helix 48–50, Helix 58–68, Helix 71–73, Helix 74–85, Beta strand 87–93, Beta strand 96–101, Helix 104–112, Helix 124–132, Helix 139–141, Helix 144–158, Helix 161–164, Helix 167–189, Beta strand 190–196, Helix 199–215, Beta strand 223–225, Helix 228–244, Helix 245–247, Helix 252–258, Helix 260–291, Helix 301–307, Helix 313–328, Helix 330–344, Helix 346–362, Turn 363–365, Helix 367–370, Helix 375–387, Beta strand 390–397, Beta strand 402–404, Beta strand 407–409, Beta strand 414–418, Helix 419–423, Turn 426–428, Beta strand 429–431, Helix 437–440, Turn 449–451, Helix 458–460, Helix 465–482, Beta strand 483–486, Beta strand 495–505, and Beta strand 509–513.

Methods

The X-ray refinement for Monte Carlo was built using YASARA SSP/PSSM Method . The structure was relaxed to the YASARA/Amber force field using knowledge-based potentials within YASARA. The side chains and rotamers were adjusted with knowledge-based potentials, simulated annealing with explicit solvent, and small equilibration simulations using YASARA’s refinement protocol .

Refinement of the finalized models was completed using either Schrodinger’s LC-MOD Monte Carlo-based module or NAMD2 protocols. These refinements started with YASARA generated initial refinement and mutant E314K . The superposition and subsequent refinement of the overlapping regions yields a complete model for Cyp11A1. The final structures were subjected to energy optimization with PR conjugate gradient with an R-dependent dielectric.

Atom consistency was checked for all 521 amino acids, verifying correctness of chain name, dihedrals, angles, torsions, non-bonds, electrostatics, atom-typing, and parameters. Each model was exported to the following formats: Maestro (MAE), YASARA (PDB). Model manipulation was done with Maestro (Macromodel, version 9.8, Schrodinger, LLC, New York, NY, 2010), or Visual Molecular Dynamics (VMD) .

Monte Carlo dynamics searching (LCMOD-MC) was completed on each model for conformational sampling, using methods previously described in the literature . Briefly, each Cyp11A system was minimized with relaxed restraints using either Steepest Descent or Conjugate Gradient PR, then allowed to undergo the MC search criteria, as shown in the literature . The primary purpose of MC, in this scenario, is examining any conformational variability that may occur with different mutation in the region near to the mutation and cholesterol-binding pocket.

**Reference:**

1. Krieger E*, et al.* (2009) Improving physical realism, stereochemistry, and side-chain accuracy in homology modeling: Four approaches that performed well in CASP8. *Proteins* 77 Suppl 9:114-122.

2. Altschul SF*, et al.* (1997) Gapped BLAST and PSI-BLAST: a new generation of protein database search programs. *Nucleic Acids Res* 25(17):3389-3402.

3. Hooft RW, Vriend G, Sander C, & Abola EE (1996) Errors in protein structures. *Nature* 381(6580):272.

4. Hooft RW, Sander C, Scharf M, & Vriend G (1996) The PDBFINDER database: a summary of PDB, DSSP and HSSP information with added value. *Comput Appl Biosci* 12(6):525-529.

5. King RD & Sternberg MJ (1996) Identification and application of the concepts important for accurate and reliable protein secondary structure prediction. *Protein Sci* 5(11):2298-2310.

6. Qiu J & Elber R (2006) SSALN: an alignment algorithm using structure-dependent substitution matrices and gap penalties learned from structurally aligned protein pairs. *Proteins* 62(4):881-891.

7. Laskowski RA, Macarthur MW, Moss DS, & Thornton JM (1993) Procheck - a Program to Check the Stereochemical Quality of Protein Structures. *J Appl Crystallogr* 26:283-291.

8. Humphrey W, Dalke A, & Schulten K (1996) VMD: visual molecular dynamics. *J Mol Graph* 14(1):33-38, 27-38.

9. Caulfield T & Devkota B (2012) Motion of transfer RNA from the A/T state into the A-site using docking and simulations. *Proteins*.

10. Caulfield T & Medina-Franco JL (2011) Molecular dynamics simulations of human DNA methyltransferase 3B with selective inhibitor nanaomycin A. *J Struct Biol* 176(2):185-191.

11. Caulfield TR, Devkota B, & Rollins GC (2011) Examinations of tRNA Range of Motion Using Simulations of Cryo-EM Microscopy and X-Ray Data. *J Biophys* 2011:219515.

12. Caulfield TR (2011) Inter-ring rotation of apolipoprotein A-I protein monomers for the double-belt model using biased molecular dynamics. *J Mol Graph Model* 29(8):1006-1014.
